# Permanent Data Storage in ZnO Thin Films by Filamentary Resistive Switching

**DOI:** 10.1371/journal.pone.0168515

**Published:** 2016-12-19

**Authors:** Adolfo Henrique Nunes Melo, Marcelo Andrade Macêdo

**Affiliations:** Department of Physics, Federal University of Sergipe, São-Cristóvão, Sergipe, Brazil; Institute of Materials Science, GERMANY

## Abstract

Resistive memories are considered the most promising candidates for the next generation of non-volatile memory; however, attention has so far been limited to rewritable memory features for applications in resistive random access memories (RRAM). In this article, we provide a new insight into the applicability of resistive memories. The characteristics of non-rewritable resistive memories (NRRM) were investigated. Devices with Pt/ZnO/ITO architecture were prepared using magnetron sputtering, upon which various bipolar and unipolar resistive switching tests were performed. The results showed excellent distinction between the high resistance state (HRS) and low resistance state (LRS), with *R*_*HRS*_/*R*_*LRS*_ = 5.2 × 10^11^ for the Pt/ZnO/ITO device with deposition time of 1 h. All samples were stable for more than 10^4^ s, indicating that the devices have excellent applicability in NRRMs.

## Introduction

Recently much attention has been paid to the new generation of non-volatile memories through applications in resistive random access memories (RRAM), which is based on controlled changes in resistance state by an applied voltage. However, there are other possibilities for data storage, such as non-rewritable resistive memories (NRRM), which display high storage density, ultra-fast recording, low consumed power, and stable switching performance with a wide window between resistance states [1]. The memory effect is based on resistive switching mechanisms that can be explained through the creation and destruction of conductive filaments in devices with metal-insulator-metal (MIM) structures [2–4]. In recent years, intensive research into resistive switching mechanisms has observed high mobility of oxygen vacancies in the crystal lattice of devices, which has an important role in the resistive switching process [1]. However, there are other models that explain resistive switching, such as modulations at Schottky-like barriers, which present a continuous and asymmetric switching induced by the application of an electric field [5], the trap-controlled space-charge-limited current (SCLC) model [6–8], and electrical polarization in materials with ferroelectric characteristics [9]. Various materials exhibit resistive switching effects, for example, transition metal oxides such as ZnO_x_, Al_2_O_3_, CuO_x_, NbO_x_, perovskites, or SrTiO_3_ and BiFeO_3_ [1,2,10]; however there are no reports on the use of resistive switching effects for permanent data storage in resistive memories.

It is known that thin films of ZnO may show unipolar resistive switching (URS) behavior in which the switching process is independent of the bias voltage polarity applied to devices [4,11–13]. Bipolar switching behavior (BRS) in ZnO thin films is achieved by changing the polarity of the applied voltage [7,14]. Some works present a coexistence with [15] or transition to unipolar behavior [1,16], associated with the maximum level of current applied to the device. Previous works with ZnO/Pt/ZnO devices grown by pulsed laser deposition (PLD) had X-ray diffraction patterns indicating high preferential orientation of ZnO thin films, in addition to optical transmission characteristics about 60% in visible range which may possibility it to electro-optical systems involving ZnO and conductors layers, such as Pt or ITO (indium tin oxide) [17]. ZnO thin films beyond memories features, are an attractive II-VI semiconductor which have a great potential in light emitting diodes and ultraviolet-blue semiconductor lasers [17,18]. Howerver, in this work, we have performed URS and BRS behavioral tests on devices built with a Pt/ZnO/ITO structure prepared with magnetron sputtering, and investigated their performance as resistive memory for permanent data storage. ITO is known to have high conductivity and high optical transmission in the visible region, which, along with the transparent layer of ZnO (band gap ~ 3.3 eV) [18–21], may favor applications for these devices in transparent electronics [7,19]. We also show that resistive memories have high potential for applications in the next generation of NRRMs, due to the high ratio between high and low resistance states. These NRRMs are an alternative for devices in which the information stored will not be lost with time and are not possible rewrite with newer information upon that ready stored, which can guarantee non modification of the data. This kind of resistive memories does not suffer stress and degradation of material occasioned by recording and rewriting of information in rewritable resistive memories.

## Experimental

ZnO thin films were grown on ITO substrates (Asahi Glass AGC) using a RF magnetron sputtering system (AJA International) at a power of 100 W on a ZnO (99.9%) ceramic target (Macashew Technology) with an Ar pressure of 20 mTorr. The deposition was performed without heating and no additional oxygen was supplied. The deposition time was varied from 30 min to 3 h. The structural analysis was carried out using X-ray diffraction (XRD) (Bruker D8 Advance). To evaluate the thickness and morphology of the devices, scanning electron microscopy (SEM) measurements of device cross sections were performed (JSM-6510LV). Dispersive X-ray Spectroscopy (EDS) (JSM-6510LV) was used to determine the composition of the thin films. Electrical and resistive switching characterizations were performed using a Pt metal tip with a diameter of ~200 *μ*m as the upper electrode; the Pt tip was applied directly to the surface of the ZnO thin film. A voltage and current supply (Keysight Agilent B2901) was used in voltage scan mode at room temperature. All operating voltages were applied to the top Pt electrode while the lower ITO electrode was kept grounded.

## Results and Discussion

**Fig 1:**
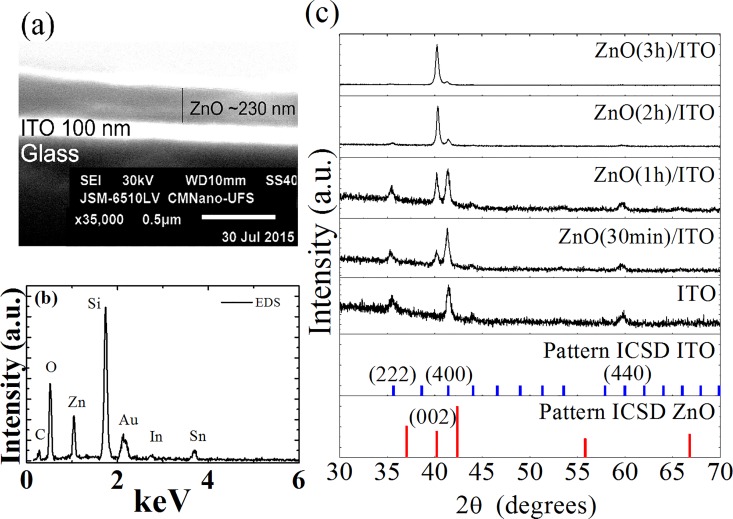
**(a) SEM images of the cross section of ZnO (30 min)/ITO. (b) EDS spectra for ZnO (30 min)/ITO. (c) XRD patterns of ZnO (*t*)/ITO samples and ITO substrates.** ZnO and ITO ICDS patterns are also shown.

Fig 1(A) shows the SEM image of the cross section for the ZnO (30 min)/ITO device. It can be observed that the thickness of the ZnO layer shows good homogeneity (~ 230 nm) and that there is a sharp ZnO/ITO interface (100 nm). The EDS spectra of ZnO (30 min)/ITO can be seen in Fig 1(B), showing the presence of Zn and O as well as Sn and In components. The Au contribution is due to a surface coating of 20 nm of gold, needed to prevent charging in these measurements. The presence of C is assigned to the vacuum conditions during the scanning process, and is common in this type of measurement. The XRD patterns of ZnO (*t*)/ITO devices with deposition time *t* = 30 min, 1 h, 2 h and 3 h are shown in Fig 1(C). The (002) diffraction peaks of the ZnO film and (222), (400) and (440) peaks of ITO were determined using the ICSD database (Inorganic Crystal Structure Database–ITO pdf number: 01-089-4596; ZnO pdf number: 01-074-0534 36–1451). ZnO thin films exhibited high orientation preference along the *c* axis with hexagonal wurtzite crystal structure, without presenting secondary phases. Earlier works on ZnO thin films showed that the thin film was textured along the perpendicular direction of substrate [17,18,21,22]. The increase in the peak intensity with deposition time of the (002) peak in ZnO thin films is associated with increased crystallinity [13,21].

**Fig 2:**
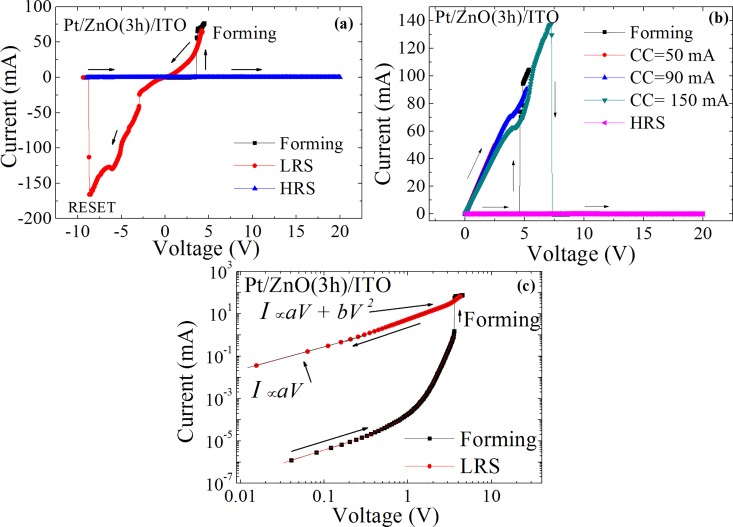
Resistive switching in Pt/ZnO (3 h)/ITO. (a) Current-voltage characteristics (*I-V*) of the bipolar test on the Pt/ZnO (3 h)/ITO device. (b) *I-V* Characteristics of the unipolar test on the Pt/ZnO (3 h)/ITO device. (c) *log-log* scale of bipolar *I-V* curves. Arrows indicate the direction of the voltage sweep.

**Fig 3:**
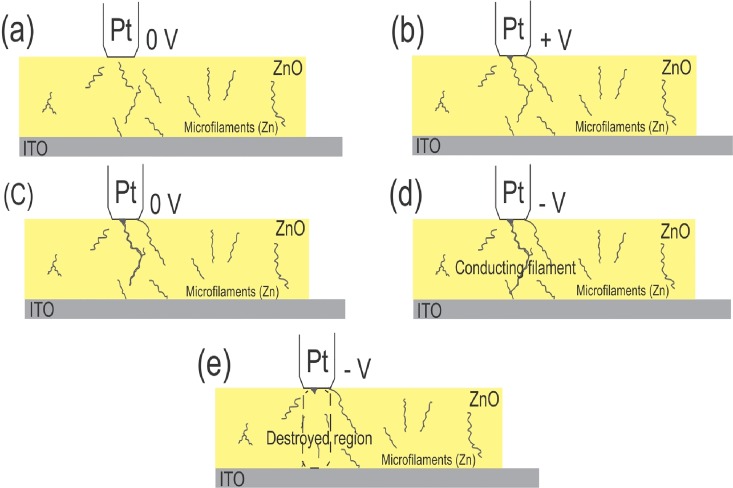
Schematic representation of forming processes and destruction of conducting filaments. (a) Preexisting filaments in the ZnO thin film. (b) When voltage is applied, some filaments connect to each other, however some regions remain where conduction is based on tunneling through preferential sites. (c) When the equipment is turned off, a robust filament has already formed. (d) Sweeping the voltage negatively causes a complete conducting path to form, connecting an electrode to another. (e) At high currents, the filament and its surroundings are damaged by Joule heating.

The current-voltage (*I-V*) characteristics for bipolar and unipolar tests of the Pt/ZnO (3 h)/ITO device are shown in Fig 2(A) and 2(B), respectively. For the bipolar test, the voltage followed the sequence: 0 V → 5 V → shutdown → 5 V → -10 V → shutdown → 20 V. Two well-defined resistance states can be observed, a high resistance state (HRS) before the forming process and a low resistance state (LRS) after forming. The memory effect can be seen after the forming process, in which the device remains in the LRS when the voltage is swept again. However, although the forming process has been observed, quadratic behavior of the current suggests that the formed conductive path is based on a coupling between filaments and trap-controlled SCLC, where the connection between filaments is a result of the tunneling of electrons through neighboring sites, explained by Child’s law (*I* ∝ *V*^2^) [8]. This effect is also seen in Fig 2(C) in a double log scale, which shows that the LRS still displays a typical quadratic behavior. Similar non-linear ohmic *I-V* characteristics at high resistance state were found in ZnO/AlN/Si(111) heterostructure thin films deposited by PLD, where this process result from enhanced excitation of charge carriers from defects levels at higher voltages [23]. Furthermore, at LRS when the voltage is swept negatively, the current shows a sudden increase at ~ -4 V, possibly due to a fusion of preexisting conducting filaments. This fusion may form a large metallic conductive path promoting a linear change in current with applied voltage. The formation of the conductive path is caused by driving defects like oxygen vacancies, which capture electrons and form conducting filaments [16]. Upon reaching currents of ~10^2^ mA, instabilities are observed due to Joule heating, with subsequent complete destruction of the filament and a switch to the HRS of the device. Excessive heating results in a destructive breakdown, after which no new forming process is observed. Fig 3 shows a forming process scheme and the resistive switching mechanism observed in our samples.

The unipolar as well as bipolar behavior, is characterized by a pronounced formation process (HRS → LRS). Two current jumps are observed in the forming process, however, opposite of what occurred in bipolar test, the second jump of the current occurred during the positive sweep voltage in agreement with the hypothesis of a connection between neighboring conducting filaments. After completed formation, the device displays Ohmic behavior in the conductive state. The gradual increase of compliance current (CC) from 50 mA to 150 mA demonstrates the persistence of the conductive state, with breakdown occurring only at high currents.

It is believed that the samples show a high level of oxygen vacancies that naturally end up forming small conductive filaments through the thin film network of ZnO (Fig 3). Therefore, resistive memory based on a single forming process is observed. With a high level of oxygen vacancies, associated with the formation of a robust conducting filament, there are insufficient amounts of oxygen available to recombine with filament Zn atoms in order to avoid permanent damage, resulting in a breakdown where the entire filamentary structure and its surroundings are damaged irreversibly [16].

**Fig 4:**
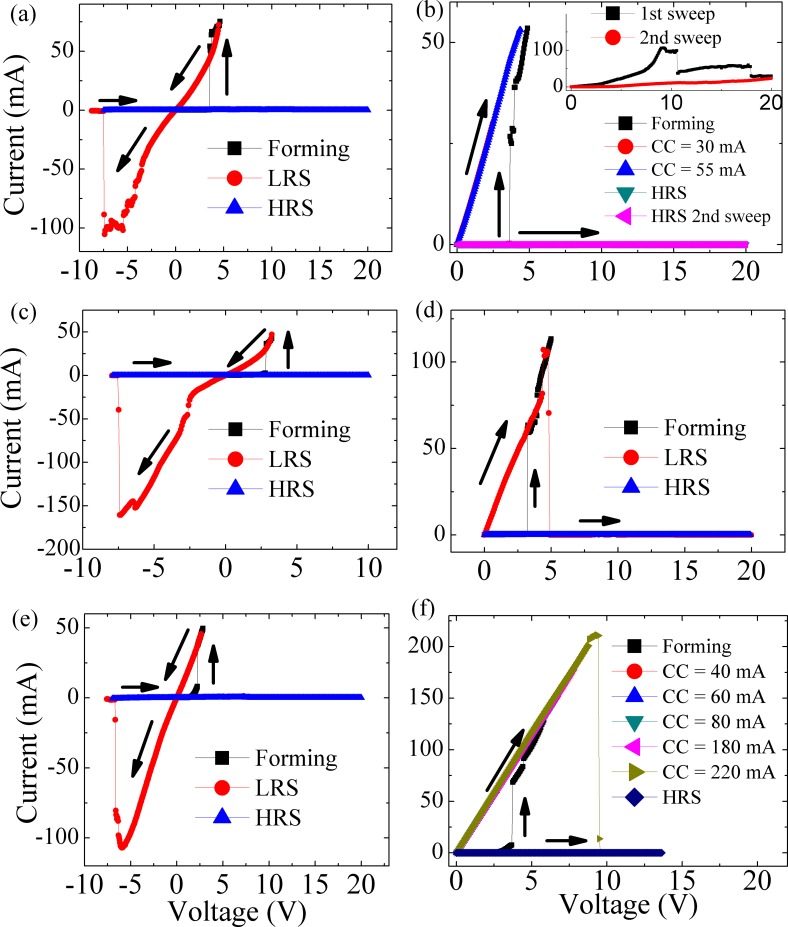
Typical *I-V* curves of Pt/ZnO (*t*)/ITO devices, with *t* = 2 h, 1 h and 30 min. Bipolar tests (a), (c) and (e) for Pt/ZnO (2 h, 1 h, 30 min)/ITO devices. Unipolar tests (b), (d) and (f) for Pt/ZnO (2 h, 1 h, 30 min)/ITO devices. Inset in (b) is an amplification of two sweeps after the RESET process.

**Fig 5:**
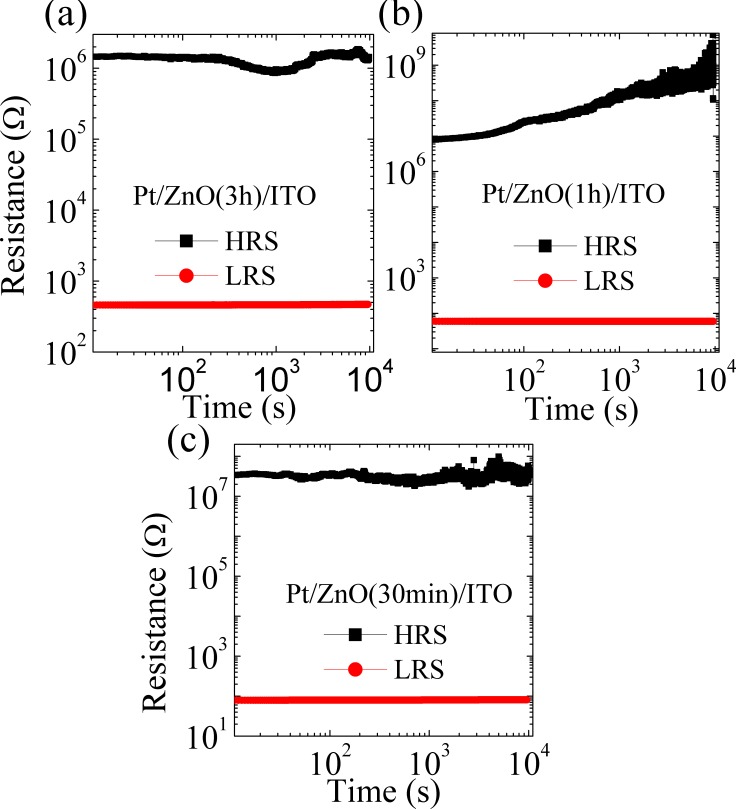
Retention curves as a function of time for HRS and LRS. (a) Pt/ZnO (3 h)/ITO, (b) Pt/ZnO (1 h)/ITO and (c) Pt/ZnO (30 min)/ITO.

Fig 4 shows the typical *I-V* curves of bipolar and unipolar testing of the Pt/ZnO (*t*)/ITO devices for t = 30 min, 1 h and 2 h. In all samples, HRS and LRS are observed after the forming process. After the RESET, samples return to the insulating state. It can be appreciated that in order to use resistive memory for permanent data storage, all devices showed resistive states well defined. Due to destructive characteristics of switching from LRS to HRS, the permanent storage is achieved. To evaluate the off/on ratio between resistance states for devices, retention curves were measured (Fig 5) using a fixed operating voltage of 50 mV. It is observed that the Pt/ZnO (1 h)/ITO device presented a memory window defined as [24] (*R*_*HRS*_ − *R*_*LRS*_)/*R*_*LRS*_∼*R*_*HRS*_/*R*_*LRS*_ resulting in a value 5.2×10^11^ for this device. Such a high off/on ratio has not been measured before for any resistive memory device [1,12,25]. This high ratio results in a faster reading speed, which when combined with the low forming voltages (~2–5 V) observed in all devices, results in improved recording ability. The HRS is stable over time and shows no overlap with the LRS. However, an increase of resistance in the HRS with time can be seen (Fig 5(B)). It is believed that the voltage of 50 mV applied to the device during testing causes a temperature increase in the filaments through the Joule effect, resulting in an increased resistance and fluctuation of the resistance measurements.

The retention curves with *t* = 3 h and 30 min for ZnO devices showed no increase in resistance with time, probably due to more pre-existing neighboring conducting filaments. More neighboring filaments imply less resistance because there are more conducting pathways for electrons to cross the ZnO lattice. However, regardless of this fact, the samples showed a high level of retention of the resistance states, proving the efficiency of the devices as NRRM. It is also observed that the Pt/ZnO (3 h)/ITO had an average off/on ratio of 3.1 × 10^8^, below the average ratio for *t* = 1 h and 30 min devices, probably due to a higher concentration of microfilaments facilitating electronic migration, leaving a less resistive sample. This result also shows the repetitively resistance state of samples which remains along to 1×10^4^ s, indicating that Pt/ZnO(*t*)/ITO may be useful to storage data. Similar results were achieved on different samples.

**Fig 6:**
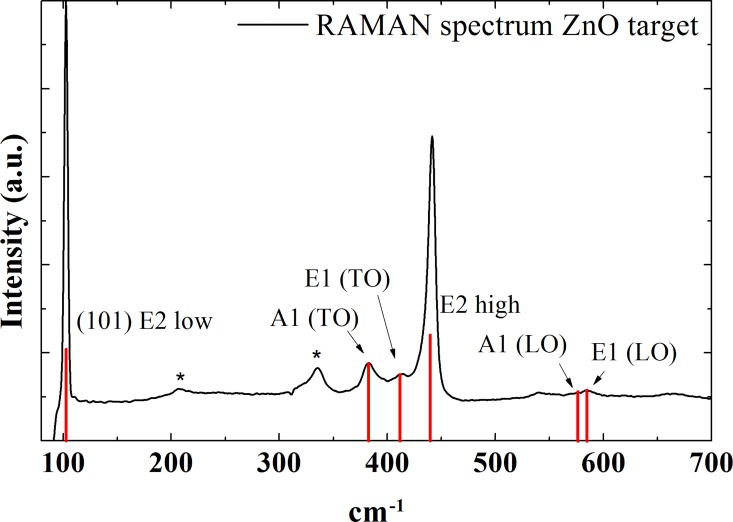
Raman spectrum of ZnO target. The Raman scattering peaks were identified with regard to vibrational modes of the ZnO network (E2 low A1 (TO), E1 (TO), E2 high A1 (LO), E1 (LO)), two Raman scattering peaks could not be identified (marked with asterisks). The red lines are included to improve clarity for the reader.

Finally, to confirm the hypothesis that excessive oxygen vacancies are responsible for the non-rewritable trait, a Raman spectrum was measured using a piece from the ZnO target that was used for film depositions (Fig 6). The Raman spectrum showed all the characteristic peaks of the main vibrational modes of the hexagonal wurtzite structure of ZnO [26,27]. The peaks for the vibrational modes A1 (LO) and E1 (LO) are attributed to the absence of oxygen or interstitial zinc (excess zinc) [26,28], This finding agrees with our argument for the presence of metallic Zn microfilaments inside the ZnO layer on the devices.

## Conclusions

In this study, the crystal and resistive switching properties of Pt/ZnO (*t*)/ITO devices with *t* = 30 min, 1 h, 2 h, and 3 h were investigated. According to XRD results, ZnO thin films were highly oriented along the *c* axis with a (002) peak increasing in intensity with ZnO deposition time. The resistive switching characteristics showed efficient operation with potential for use in developing non-rewritable resistive memory for permanent storage of data. Bipolar behavior on all devices presented transitions from HRS to LRS with clearly defined resistive states. Unipolar tests indicated that increasing the upper current limit did not result in non-destructive RESET, which confirms the NRRM characteristic of the samples. Both bipolar and unipolar behavior were explained by the creation and destruction of conducting filaments, where it is believed that there are microfilaments previously formed in the ZnO network due to a high concentration of oxygen vacancies. The retention tests showed excellent stability for LRS and HRS with no indication of state overlap, with a maximum memory window of 5.2 × 10^11^ for the Pt/ZnO (1 h)/ITO device. In addition, the results revealed a new insight into applying resistive switching in NRRMs.
